# Mobilization of lipids and fortification of cell wall and cuticle are important in host defense against Hessian fly

**DOI:** 10.1186/1471-2164-14-423

**Published:** 2013-06-26

**Authors:** Chitvan Khajuria, Haiyan Wang, Xuming Liu, Shanda Wheeler, John C Reese, Mustapha El Bouhssini, R Jeff Whitworth, Ming-Shun Chen

**Affiliations:** 1 Department of Entomology, Kansas State University, Manhattan 66506, Kansas; 2 Department of Statistics, Kansas State University, Manhattan 66506, Kansas; 3 International Center for Agricultural Research in the Dry Area, Allepo, Syria; 4 Hard Winter Wheat Genetics Research Unit, USDA-ARS, Kansas State University, 4008 Throckmorton, Manhattan 66506, Kansas; 5 Current Address: Department of Entomology, University of Nebraska, Lincoln 68583, Nebraska

## Abstract

**Background:**

Wheat – Hessian fly interaction follows a typical gene-for-gene model. Hessian fly larvae die in wheat plants carrying an effective resistance gene, or thrive in susceptible plants that carry no effective resistance gene.

**Results:**

Gene sets affected by Hessian fly attack in resistant plants were found to be very different from those in susceptible plants. Differential expression of gene sets was associated with differential accumulation of intermediates in defense pathways. Our results indicated that resources were rapidly mobilized in resistant plants for defense, including extensive membrane remodeling and release of lipids, sugar catabolism, and amino acid transport and degradation. These resources were likely rapidly converted into defense molecules such as oxylipins; toxic proteins including cysteine proteases, inhibitors of digestive enzymes, and lectins; phenolics; and cell wall components. However, toxicity alone does not cause immediate lethality to Hessian fly larvae. Toxic defenses might slow down Hessian fly development and therefore give plants more time for other types of defense to become effective.

**Conclusion:**

Our gene expression and metabolic profiling results suggested that remodeling and fortification of cell wall and cuticle by increased deposition of phenolics and enhanced cross-linking were likely to be crucial for insect mortality by depriving Hessian fly larvae of nutrients from host cells. The identification of a large number of genes that were differentially expressed at different time points during compatible and incompatible interactions also provided a foundation for further research on the molecular pathways that lead to wheat resistance and susceptibility to Hessian fly infestation.

## Background

In addition to constitutive factors, plants may also launch chemical defense in response to herbivore attack. Two types of plant defenses have been reported: basal defense (or innate immunity) and resistance (R) gene-mediated defense (or induced defense) [1]. Basal defense is present in all plants and is triggered by a general perception of parasite-derived general elicitors (similar to pathogen-associated molecular patterns, PAMPs) [2]. Suppression of basal defense leads to plant susceptibility and parasite establishment. R gene-mediated defense exists only in plants with an effective R gene and is triggered by a specific recognition between a plant R protein and a parasite avirulence (Avr) effector [3,4]. The recognition of parasite effectors in both basal and R gene-mediated defenses lead to the production of the signaling molecules such as salicylic acid (SA), jasmonic acid (JA), nitric oxide, ethylene, and various polyunsaturated fatty acids (PUFAs) [5-10]. These signaling molecules trigger cascades of signal transduction pathways, leading to the launch of toxic chemical defenses and a transient reduction in primary metabolism [11].

In recent decades, rapid advances have been achieved in elucidation of molecular processes in plant defense. Numerous plant resistance genes have been cloned and many key components in defense signaling pathways have been identified [12-14]. The characterization of resistance genes and signaling molecules has greatly enriched our understanding of plant defenses at the molecular level. In addition, global approaches including microarrays and metabolite profiling have also been adapted to elucidate changes in metabolic pathways that result in plant resistance or susceptibility in response to herbivore attack [15,16]. Despite these advances, however, our understanding of molecular events in plant defense is far from comprehensive. Most of our understanding of plant defense is from studies of plant-pathogen interactions [17]. Little is known regarding the molecular pathways that lead to plant defense against insects, especially plant resistance to piercing-sucking insects [18]. Only two resistance genes conferring plant resistance to insects have been molecularly identified so far, the tomato *Mi-1.2* gene, which has been found to confer resistance to some isolates of *Macrosiphum euphorbiae* (potato aphid) and *Bemisia tabaci* (silverleaf whitefly) [19,20]; and the virus aphid transmission (*Vat*) gene, which has been found to control resistance to *Aphis gossypii* (cotton aphid) [21]. Global analysis of changes in gene expression in host plants following insect attack has also been limited to a few plant – insect systems [22-27].

The Hessian fly (*Mayetiola destructor*) is a member of a large group of insects called gall midges and can be a destructive pest of wheat [28]. Hessian fly larvae live between leaf-sheaths of a wheat plant. Unlike other gall midges which induce the formation of outgrowth galls on plants, a Hessian fly larva converts the whole susceptible plant into a gall by inducing the formation of nutritive cells and a nutrient sink at the feeding site just above the base of a wheat seedling, and by inhibiting plant growth [29,30]. Failure to induce formation of nutritive cells in plants results in the death of Hessian fly larvae, as seen in resistant wheat plants that contain an effective resistance gene [30]. Wheat resistance to Hessian fly is controlled by major dominant resistance genes that exhibit a typical gene-for-gene relationship with insect avirulence [28]. Several studies have been carried out to investigate genes that are likely involved in plant resistance, including genes encoding lectin-like proteins [31], proteinase inhibitors [32], enzymes involved in cell wall metabolism [33], enzymes involved in the production of reactive oxygen species [34], enzymes involved in primary metabolism [11], and proteins involved in lipid metabolism [35,36]. These studies on specific groups of genes have enhanced our understanding of wheat – Hessian fly interactions. A more systematic approach to examine global changes in gene expression in wheat plants following Hessian fly attack is also needed to reveal molecular pathways of wheat defense against this pest.

An initial study using DNA microarray identified numerous genes that are either up- or down-regulated in plants during incompatible and compatible interactions after 72 h following Hessian fly infestation [33]. Recently, we found that wheat defense responses to Hessian fly attack occur much earlier and more rapidly than originally thought [36]. Early response genes are most likely to determine wheat resistance or susceptibility to Hessian fly infestation. The objectives of this study were 1) to conduct global analyses of gene expression using microarrays and other tools to identify early response genes in resistant plants during incompatible interactions, 2) to identify early response genes in susceptible plants during compatible interactions, and 3) to identify key pathways that are crucial for plant resistance by comparing the dynamic differences of gene expression and metabolite accumulation during incompatible and compatible interactions. Our results indicate that a combination of rapid resource mobilization, elevated toxic chemicals, and cell wall fortification at the early stage plays a central role in wheat resistance to the Hessian fly.

## Results

### Hessian fly induces rapid and large scale changes in wheat gene expression

**Figure 1:**
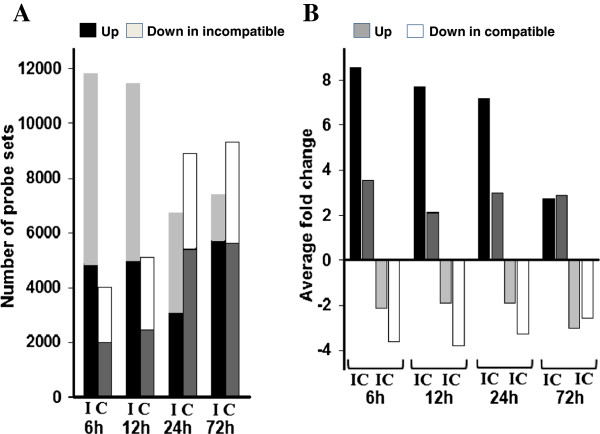
**Hessian fly infestation induces large scale of changes in gene expression in wheat seedlings. A**: Numbers of probe sets that detected statistically significant (*P* < 0.05) changes in transcript abundance in wheat seedlings at 6, 12, 24, and 72 h following Hessian fly infestation. The letters ‘I’ and ‘C’ represent resistant plants during ‘incompatible’ interactions and susceptible plants during ‘compatible’ interactions, respectively. **B**: Average fold changes of transcript abundance in plants attacked by Hessian fly. Denotations are the same as in panel A.

To assess the impact of Hessian fly infestation on wheat gene expression, Affymetrix wheat microarrays were used to identify up- and down-regulated genes in resistant plants during incompatible interactions, and in susceptible plants during compatible interactions, respectively, following Hessian fly infestation. Microarray data were then validated through quantitative real-time PCR (qPCR) (see Methods). As shown in Figure 1A, a large number of probe sets detected significant changes in transcript abundance in both resistant and susceptible plants following Hessian fly infestation. However, the trends of the changes were different between resistant and susceptible plants. In resistant plants, many more probe sets detected significant changes in transcript abundance at 6 and 12 h than at 24 and 72 h. Just the opposite occurred in susceptible plants; more probe sets detected significant changes in transcript abundance at 24 and 72 h than at 6 and 12 h. Specifically, 11767, 11447, 6708, and 7443 probe sets, representing 19.2, 18.7, 10.9, and 12.1% of the total 61,127 probe sets contained in the microarray, detected statistically significant (*P* ≤ 0.05) changes in transcript abundance in resistant plants at 6, 12, 24, and 72 h, respectively (Figure 1A). In comparison, 4014, 5123, 8912 and 9341, representing 6.5, 8.3, 14.5, and 15.2% of the total probe sets, detected significant changes in transcript abundance at 6, 12, 24 and 72 h, respectively, in susceptible plants following Hessian fly infestation. Most probe sets that detected changes significant at *P* ≤ 0.05 also detected changes significant at *P* ≤ 0.01 (Additional file 1: Table S1).

Another difference between resistant and susceptible plants is the numbers of probe sets that detected down-regulation in comparison with those that detected up-regulation (Figure 1A). In resistant plants, at least 30% more probe sets detected down-regulation compared with those that detected up-regulation at earlier time points (6 and 12 h), and roughly equal or less numbers of probe sets detected down-regulation at later time points (24 and 72 h). In comparison, in susceptible plants, the numbers of probe sets detected down-regulation were roughly equal or less than those that detected up-regulation at all time points (Figure 1A).

For up-regulated transcripts, much larger magnitude changes were observed in resistant plants at earlier time points (Figure 1B). Specifically, an average increase of 8.6, 7.7, and 7.2 fold was observed at 6, 12, and 24 h, respectively, but only 2.7 fold at 72 h in resistant plants following Hessian fly infestation. In comparison, there was no great difference in up-regulated transcripts at different time points in susceptible plants. Specifically, 3.6, 2.2, 3.0, and 2.9 average fold of up-regulation were observed in susceptible plants at 6, 12, 24, and 72 h. For down-regulated transcripts, an average of about 2 fold (2.12, 1.91, 1.91) down-regulation was at 6, 12, 24 h and about 3 fold down-regulation at 72 h in infested resistant plants. In comparison, a bigger down-regulation was observed at earlier time points in infested susceptible plants. Approximately 4 fold down-regulation was observed at 6, 12, and 24 h, and about 3 fold at 72 h (Figure 1B).

### Early response genes differ from late response genes

**Figure 2:**
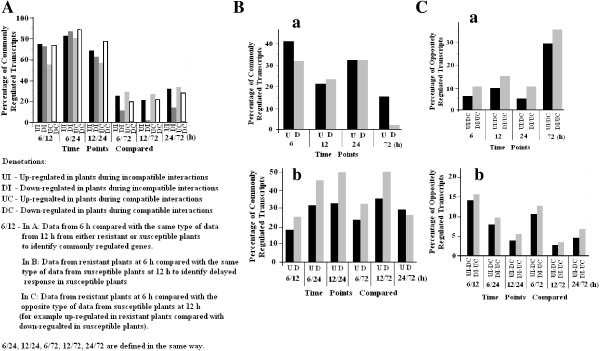
**Gene sets affected by Hessian fly at early and late time points, and in resistant and susceptible plants. A**: Gene sets in plants affected by Hessian fly at early time points (24 h or earlier) were different from those at late (72 h) time point. UI, DI, UC, and DC represent up-regulated in plants during incompatible interactions, down-regulated during incompatible interactions, up-regulated during compatible interactions, and down-regulated during compatible interactions. Commonly regulated transcripts were defined as those that were either up- or down-regulated at the two time points under comparison. Comparisons were made between time points 6 and 12 h (6/12), 6 and 24 h (6/24), 6 and 72 h (6/72), 12 and 72 h (12/72). **B**: Gene sets up-regulated in resistant plants were different from those up-regulated in susceptible plants. (**a**) Commonly up- (U) or down-regulated (D) in both resistant and susceptible plants at the same time points. (**b**) Commonly up- (U) or down-regulated (D) transcripts in resistant plants at an earlier time point and in susceptible plants at a later time point to examine potential delayed reaction in susceptible plants. **C:** Genes regulated in opposite directions in infested resistant and susceptible plants. (**a**) Percentages of affected transcripts that exhibited changes in abundance in opposite directions at the same time points. UI-DC – Percentages of up-regulated in resistant plants during incompatible interactions, which were down-regulated in susceptible plants during compatible interactions; DI-UC – Percentages of down-regulated transcripts in plants during incompatible interactions, which were up-regulated in plants during compatible interactions. (**b**) Percentages of affected transcripts with changes in opposite directions when data from infested resistant plants at an earlier time point compared with data from infested susceptible plants at a later time point.

To examine if similar or different gene sets in wheat were affected at different time periods during Hessian fly infestation, we analyzed if genes up- or down-regulated at one time point were also up- or down-regulated at a different time point. The genes that were regulated in the same direction, namely up-regulated in both time points under comparison or down-regulated in both time points, were referred to as ‘commonly regulated’. Higher percentages of commonly regulated transcripts were observed when data from two early time points were compared, whereas lower percentages of commonly regulated transcripts were observed when data from an early time point were compared with data from the late 72 h time point (Figure 2A, Additional file 2: Table S2). Specifically, in resistant plants, 74.8, 82.8, and 68.8% of the affected transcripts were commonly up-regulated when data from 6 and 12 h, 6 and 24 h, and 12 and 24 h, respectively, were compared; whereas 72.9, 86.9, and 62.7% were commonly down-regulated at these time-point combinations. In susceptible plants, 55.2, 80.6, and 56.8% of the affected transcripts were commonly up-regulated when data from 6 and 12 h, 6 and 24 h, and 12 and 24 h, respectively, were compared; and 73.9, 88.5, and 77.6% were commonly down-regulated at these respective time point combinations. In contrast, fewer than 35% of the affected transcripts were commonly regulated when data from an earlier time point were compared with data from the late 72 h time point in either resistant or susceptible plants. The biphasic nature of the results shown in Figure 2A suggested that early response genes (early genes) differ from late response genes (late genes) in both infested resistant and susceptible plants.

### Genes affected by Hessian fly in resistant plants differ from those in susceptible plants

To determine if wheat gene sets affected by Hessian fly in resistant plants were similar or different from those affected in susceptible plants, we analyzed the percentages of transcripts that were commonly regulated (either commonly up- or commonly down-regulated) in both resistant and susceptible plants at the same or different time points. As shown in Figure 2Ba, less than 42% of the affected transcripts were commonly regulated in both resistant and susceptible plants at any given time, indicating that the majority of the genes affected by Hessian fly in resistant plants were different from those in susceptible plants.

Since responses to a pathogen’s attack are often delayed in susceptible plants [9,37], we further analyzed if delayed responses were the reason for the low percentages of commonly regulated transcripts by comparing data from resistant plants at an earlier time with the data from susceptible plants at a later time. The results revealed a different pattern between up-regulated and down-regulated transcripts (Figure 2Bb, Additional file 2: Table S2). Specifically, 17 to 36% of the transcripts up-regulated in resistant plants at an earlier time were also up-regulated in susceptible plants at a later time. For down-regulated transcripts, higher percentages, 25 to 51%, of the transcripts down-regulated in resistant plants at an earlier time were also down-regulated in susceptible plants at a later time. These results suggest that there was little delayed response associated with gene up-regulation, but delayed response was more significant with genes down-regulated in susceptible plants.

We also analyzed the percentages of transcripts that were regulated in opposite directions in resistant and susceptible plants (Figure 2C). At the first 24 h, 5-16% of the transcripts up-regulated in resistant plants were down-regulated in susceptible plants, and vice versa (Figure 2Ca). At 72 h, 29.3% of transcripts up-regulated in resistant plants were down-regulated in susceptible plants; and 35.7% of transcripts down-regulated in resistant plants were up-regulated in susceptible plants. We also compared data from resistant plants at an earlier time point with data from susceptible plants at a later time point (Figure 2Cb). There appears to be no significant delayed response in terms of oppositely regulated genes in resistant and susceptible plants during the test period. Overall, results suggest that wheat gene sets affected by Hessian fly infestation in resistant plants were quite different from those affected in susceptible plants.

### Defense genes are expressed consistently at higher levels in infested resistant plants

To examine what gene types were differentially regulated between resistant and susceptible plants, we analyzed the functional categories of the affected (up- or down-regulated) genes. Most of the affected genes could not be annotated and therefore their functions were unknown. Genes with known functions were classified into 11 categories: genes involved in direct toxic defense (direct defense), genes involved in lipid metabolism (lipid metabolism), genes involved in phenylpropanoid metabolism (phenylpropanoid), genes involved in cell wall and cuticle metabolism (cell wall), genes involved in reduction/oxidation (redox), genes encoding proteases (proteases), genes involved in regulation (regulation), genes encoding structural proteins (structure), genes involved in nutrient metabolism (nutrition), genes involved in stress response (stress response), and genes with other functions (Additional file 3: Table S3).

To identify genes that may play important roles in wheat defense against Hessian fly, we examined the ratio between the relative transcript abundance in resistant plants during incompatible interactions and those in susceptible plants during compatible interactions (defined as incompatible/compatible ratio, I/C ratio) (Additional file 4: Figure S1). A positive I/C ratio (above the line pointed by the red arrow in a graph) indicates a higher level of transcript in resistant plants in comparison with that in susceptible plants, whereas a negative I/C ratio indicates a lower level of transcript in resistant plants compared with that in susceptible plants. Even though each gene category exhibited quite a different pattern of I/C ratio distribution, some gene categories share certain degree of similarity. For the gene categories of ‘direct defense’, ‘lipid metabolism’, ‘phenylpropanoid’, ‘cell wall’, and ‘redox’, the majority of transcripts exhibited a positive I/C ratio, especially during the first 24 h. These gene categories have been reported to be involved in plant defense [1,38-40]. For ‘protease’ genes, the numbers of transcripts exhibited higher or lower I/C ratio were roughly the same. However, many transcripts encoding cysteine proteases exhibited very high levels of abundance during the first 24 h in resistant plants, making the graph asymmetrical towards higher levels of transcript abundance in resistant plants (Additional file 4: Figure S1). Genes encoding cysteine proteases have been reported to be induced in plants for toxic defense [41,42]. For the other gene categories including ‘regulation’, ‘structure’, ‘nutrition’, and ‘stress response’, roughly equal numbers of transcripts exhibited positive and negative I/C ratio. Clearly, defense-related genes, including genes involved in direct defense, lipid metabolism, phenylpropanoid metabolism, cell wall and cuticle metabolism, and redox were expressed at higher levels in resistant plants than those in susceptible plants, whereas other categories of genes exhibited no significant differences in expression levels in infested resistant and susceptible plants.

### Genes for resource mobilization are up-regulated rapidly in infested resistant plants

Rapid mobilization and re-utilization of resources is a necessary process for plant defense, and involves catabolic enzymes and transporters. The largest group among the genes encoding catabolic enzymes and transporters were ‘lipid metabolism’ genes encoding lipases, lipid transfer proteins, and other down-stream lipid-catabolic enzymes (Additional file 5: Table S4). Over 72% of the 182 transcripts of the ‘lipid’ genes were up-regulated in infested resistant plants, whereas less than 28% of the transcripts were down-regulated in these plants (Additional file 6: Figure S2Aa). In contrast, less than 32% of these transcripts were up-regulated in susceptible plants at 24 h or earlier, but increased to ~50% at 72 h. The magnitudes of up-regulation in resistant plants were much stronger as well (Additional file 6: Figure S2Ab). Specifically, the average fold changes of these ‘lipid’ genes were 21.6, 17.6, 8.8, and 3.6, respectively at 6, 12, 24, and 72 h, compared with 1.7, 1.4, 2.3, and 2.4 average fold increases in susceptible plants during the same period. Analyses of 164 transcripts encoding other types of transporters (Additional file 6: Figure S2B) and 52 transcripts encoding other catabolic enzymes involved in carbohydrate and amino acid metabolism (Additional file 6: Figure S2C) revealed a similar trend, higher percentages of up-regulated transcripts with greater magnitude of up-regulation at earlier time points (6 and 12 h) in infested resistant plants compared with susceptible plants. No such trend was observed in a similar analysis of 41 transcripts encoding various anabolic enzymes (Additional file 6: Figure S2D).

**Figure 3:**
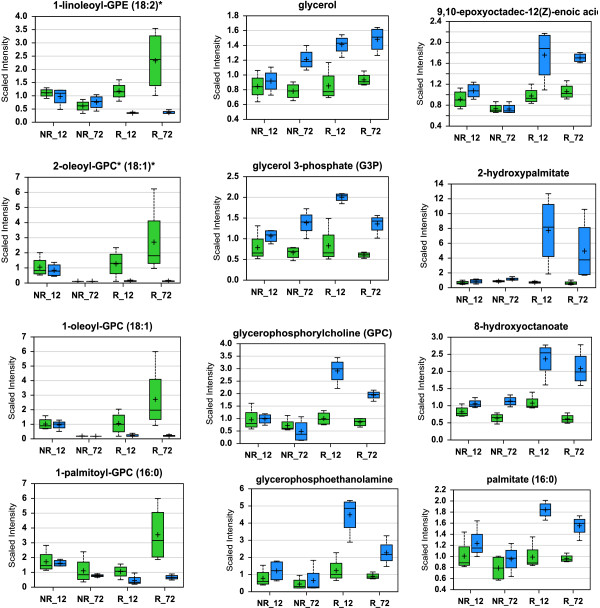
**Changes in phospholipids and related metabolites indicate rapid and extensive membrane remodeling and lipid release in infested resistant plants.** The ordinate of a graph shows the relative intensity (scaled intensity) of an intermediate in MS detection. NR_12 and NR_72 in the abscissa indicate samples from non-resistant wheat plants (susceptible Newton) at 12 and 72 h, respectively, after Hessian fly infestation, whereas R_12 and R_72 indicate samples from resistant plants (Molly seedlings) at these two time points. Green bars in a graph indicate un-infested controls, whereas blue bars indicate infested samples. Within a bar, the symbol ‘+’ indicates mean value and the symbol ‘▯’ indicates median value. The top and bottom boundaries of a bar represent upper quartile and lower quartile, respectively. The upper and lower lines above and below a bar indicate maximum and minimum distributions, respectively. The graphs on the left side of the figure show decreased abundance of four membrane lipids in infested resistant plants. The four lipids are 1-linoleoyl-glycerophosphoethamine (1-linoleoyl-GPE) (18:2), 2-oleoyl-glycerophosphocholine (2-oleoyl-GPC) (18:1), 1-oleoyl-glycerophosphocholine (1-oleoyl-GPC) (18:1), and 1-palmitoyl-glycerophosphocholine (1-palmitoyl-GPC) (16:0). The graphs in the middle show increased abundance of four intermediates in the lipid metabolic pathway, including glycerol, glycerol 3-phosphate (G3P), glycerophosphoethanolamine, and glycerophosphoyrlcholine (GPC). The graphs on the right show increased abundance of four fatty acids or their derivatives in infested resistant plants, including 9,10-epoxyoctadec-12(z)-enoic acid, 2-hydroxypalmitate, 8-hydroxyoctanoate, and palmitate (16:0). More data are given in Table S5.

The greater proportion and higher magnitudes of up-regulated genes encoding catabolic enzymes and transporters in resistant plants suggest that certain types of substances include lipids, carbohydrates, and proteins/amino acids were mobilized for plant defense. Since lipid-related transcripts were the largest group of changes and with the highest average fold of increases, we measured metabolites of lipid metabolism in control and infested wheat seedlings. The changes in lipid metabolites suggested rapid mobilization of membrane lipids and extensive membrane remodeling in resistant plants following Hessian fly infestation (Figure 3). A significant reduction of certain membrane lipids was observed predominantly in resistant plants, including 1-linoleoyl-glycerophosphoethamine (1-linoleoyl-GPE) (18:2), 2-oleoyl-glycerophosphocholine (2-oleoyl-GPC) (18:1), 1-oleoyl-glycerophosphocholine (1-oleoyl-GPC) (18:1), 1-palmitoyl-glycerophosphocholine (1-palmitoyl-GPC) (16:0), 1-palmitoyl-glycerophosphoethamine (1-palmitoyl-GPE) (16:0), 1-palmitoyl-glyerophosphoinositol (1-palmitoyl-GPI) (16:0), and 1-palmitoylglyerol (16:0) (Figure 3 and Additional file 7: Table S5). In association with membrane lipid reduction, the abundance of a range of fatty acids and derivatives, including 9,10-epoxyoctadec-12(z)-enoic acid, 2-hydroxypalmitate, 8-hydroxyoctanoate, palmitate (16:0), oleate (18:1n9), vaccinate (18:1n7), and eicosenoate (20:1n9 or n11), was elevated specifically in resistant plants (Figure 3, Additional file 7: Table S5). In addition, the intermediates in phospholipid metabolic pathways including glycerol, glycerol 3-phosphate (G3P), glycerophosphoethamine, and glycerophosphorylcholine (GPC), were elevated predominantly in infested resistant plants. The reduction in the abundance of membrane lipids and the elevation of fatty acids and other intermediates indicated that membrane lipids were mobilized for defense.

### Direct toxic defense genes are up-regulated in infested resistant plants

**Figure 4:**
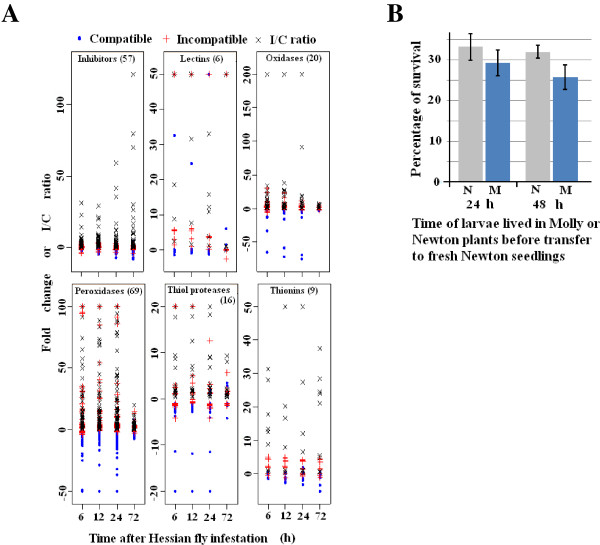
**The majority of affected transcripts encoding proteins toxic to insects or enzymes that can produce toxic chemicals are up-regulated in resistant wheat plants. A**: Transcripts encoding proteins for direct defense are up-regulated in resistant plants (red +), but down-regulated, not affected, or up-regulated to a less magnitude in susceptible plants (blue •) after Hessian fly infestation. The differential regulation of these transcripts between infested resistant and susceptible plants resulted in much higher expression levels, as measured by incompatible/compatible ratio (I/C ratio, indicated by the symbol x in the figure), in resistant plants. **B**: Toxic chemicals alone do not result in immediate lethality of Hessian fly larvae. Larvae fed in resistant plant for 24 and 48 h, respectively, were washed out from the plant after removing outside layers of leaf-sheaths. The larvae were then transferred onto new, susceptible seedlings individually. Two weeks after the transfer, the new susceptible plants were dissected to examine if the larva put on the plant was rescued (live). For control, larvae fed on susceptible Newton plants for 24 and 48 h, respectively, were washed out from dissected plants, and were then transferred to new Newton plants in exactly the same way. Statistical analysis indicated no significant difference (*P* = 0.2) between survival rates of Hessian fly larvae initially fed in Molly seedlings and those fed on Newton for either 24 or 48 hours.

Membrane remodeling and mobilization of lipids release resources that are likely used for defense against Hessian fly attack. The types of defenses that are essential for the death of Hessian fly larvae remain to be determined. Transcripts encoding proteins that are toxic or enzymes that can produce toxic chemicals to insects exhibited increased abundance in infested resistant plants. These transcripts encode 57 proteinase inhibitors, six lectins, 20 oxalate oxidases, 69 peroxidases, 16 defense proteases (secreted cysteine proteases), and nine thionins/defensins (Figure 4, Additional file 8: Table S6). Proteinase inhibitors, lectins, cysteine proteases, and thionins have been previously reported as potential toxic chemicals against Hessian fly and other insect pests in different crops [18,31,32,39,42,43]. Oxalate oxidases and peroxidases catalyze the production of hydrogen peroxide, which is toxic to pathogens and insects [34,44]. In addition, secondary metabolites produced by elevated expression of genes involved in phenylpropanoid pathways (see below).

Hessian fly larvae die in resistant plants after 72 to 96 h [29]. To determine if toxic chemicals are sufficient for causing the death of Hessian fly larvae, a rescue assay was performed with larvae fed on resistant plants for 24 and 48 h, respectively. Hessian fly larvae that had fed in resistant Molly plants were washed off after dissecting the plants, and the larvae were then put back individually onto a new, susceptible Newton plant to determine if larvae fed in resistant plants could be rescued by shifting to susceptible plants. As shown in Figure 4B, larvae fed in resistant plants can be rescued by transferring them onto susceptible plants. No statistically significant difference was observed between larvae fed on resistant Molly and control larvae fed on susceptible Newton for the same time periods before being transferred to a new susceptible plant. The results suggest that toxic defense alone in resistant plants did not cause immediate lethality of Hessian fly larvae.

### Up-regulation of ‘phenylpropanoid’ genes are associated with increased accumulation of phenylpropanoids in infested resistant plants

**Figure 5:**
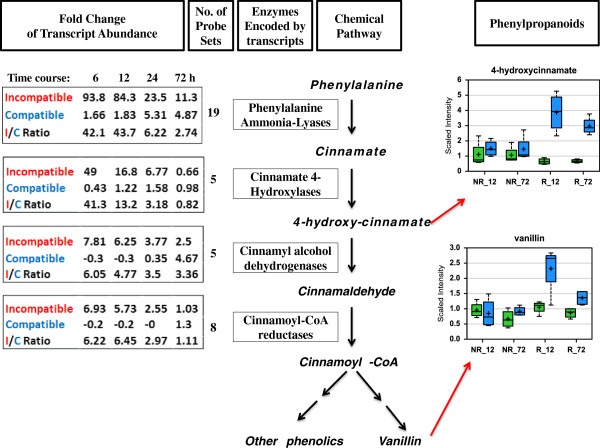
**Up-regulation of phenylpropanoid-related genes is associated with elevated accumulation of phenylpropanoids in infested resistant plants.** The chemical pathway for converting phenylalanine into various phenylpropanoids is shown in the middle of the figure along with the enzyme in each step. The fold changes in abundance of transcripts encoding these respective enzymes are shown in the left along with the number of probe sets that detected the changes. The relative concentration of two representative phenylpropanoids, 4-hydroxy-cinnamate and vanillin, are shown in the two graphs on the right side of the figure. For the two graphs, the ordinate shows the relative intensity (scaled intensity) of the intermediate in MS detection. NR_12 and NR_72 in the abscissa indicate samples from non-resistant wheat plants (susceptible Newton) at 12 and 72 h, respectively, after Hessian fly infestation, whereas R_12 and R_72 indicate samples from resistant plants (Molly seedlings) at these two time points. Green bars in a graph indicate un-infested controls, whereas blue bars indicate infested samples. Within a bar, the symbol ‘+’ indicates mean value and the symbol ‘—’ indicates median value. The top and bottom boundaries of a bar represent upper quartile and lower quartile, respectively. The upper and lower lines above and below a bar indicate maximum and minimum distributions, respectively.

A large number of probe sets detected higher levels of transcripts encoding various enzymes in the phenylpropanoid metabolic pathway in infested resistant plants (Additional file 3: Table S3). The first four chemical reactions in the phenylpropanoid pathway produce the common intermediate cinnamoyl-CoA, which is then converted into various phenylpropanoids through down-stream pathways (Figure 5). The enzymes involved in these chemical reactions include phenylalanine ammonia-lyases, cinnamate 4-hydroxylases, cinnamyl alcohol dehydrogenases, and cinnamoyl-CoA reductases. Transcripts encoding these enzymes were strongly up-regulated in resistant plants, especially at 6 and 12 h after Hessian fly infestation. In most cases, no or slight up-regulation of these transcripts was observed in infested susceptible plants. The most dramatic up-regulated transcripts were the ones encoding for phenylalanine ammonia-lyases. For the 19 transcripts encoding phenylalanine ammonia-lyases, transcript abundance increased 93.8, 84.3, 23.5, and 11.3 fold at 6, 12, 24, and 72 h, respectively, in resistant plants during incompatible interactions, and only 1.66, 1.83, 5.31, and 4.87 fold at the same period of time in susceptible plants during compatible interactions, giving I/C ratios 42.1, 43.7, 6.22, and 2.74, respectively. Transcripts encoding cinnamate 4-hyroxylases, cinnamyl alcohol dehydrogenases, and cinnaoyl-CoA-reductases were also up-regulated 2–49 fold in infested resistant plants at 6, 12, and 24 h; whereas no significant change or down-regulation was observed in infested susceptible plants.

The strong up-regulation of transcripts indicated elevated production of phenylpropanoids. The abundance of two representative phenylpropanoids, 4-hydroxy-cinnamate and vanillin, was determined using mass spectrometry (Figure 5). Both were significantly elevated in infested resistant plants, especially at 12 h. No significant changes were observed in susceptible plants during the same time period.

### Up-regulation of cell wall genes is associated with epidermal impermeability of wheat cells to the neutral red dye in infested resistant plants

**Figure 6:**
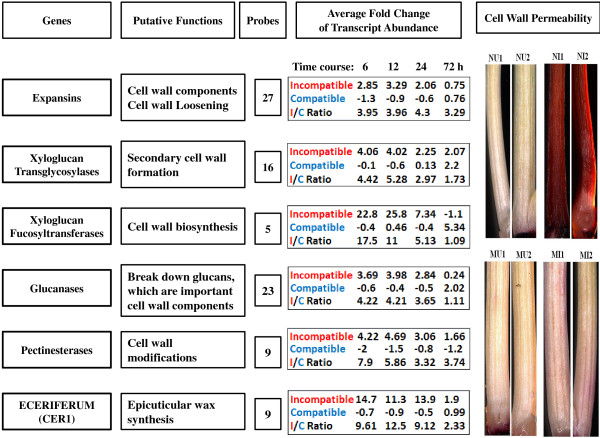
**Up-regulation of cell wall and cuticle genes is associated with epidermal impermeability of wheat plants to the neutral red dye in infested resistant plants.** The names of the genes and their putative functions are given on the left part of the figure. Fold changes and I/C ratio in transcript abundance are shown in the middle of the figure along with the numbers of probe sets that detected the changes. I/C ratio were defined as in Additional file 4: Figure S1. Results of neutral red staining for epidermal cell permeability are shown on the right. For the neutral red staining panels, NU1 and NU2 are two representatives of un-infested, susceptible Newton plants, whereas NI1 and NI2 are two Newton plants infested with Hessian fly. No staining was observed in the un-infested plants, but very strong staining was observed in infested Newton plants. MU1 and MU2 are two representatives of un-infested Molly plants, whereas MI1 and MI2 are two infested Molly plants. No staining was observed in the un-infested Molly plants, and no or very weak staining was observed in infested Molly plants.

The majority of transcripts encoding enzymes directly involved in cell wall and cuticle metabolism, including 27 expansins, 16 xyloglucan endotransglycosylases, five xyloglucan fucosyltransferases, 23 glucanases, nine pectinesterases, and nine eceriferum (CER1) exhibited higher levels of abundance in infested resistant plants (Figure 6, Additional file 3: Table S3). Expansins are cell wall components participating in cell wall loosening [45]. Xyloglucan endotransglycosylases and other enzymes are involved in various processes of cell wall biogenesis [46]. Transcripts encoding these proteins and enzymes were up-regulated on average at least 3 fold in resistant plants at 6 and 12 h after Hessian fly infestation. In comparison, the abundance of the transcripts decreased, did not change, or was slightly up-regulated at the same time period in infested susceptible plants.

To examine if elevated expression of cell wall-related genes correlates with cell wall fortification in resistant plants following Hessian fly attack, neutral red staining of cell wall penetration was conducted. In susceptible Newton plants following Hessian fly attack, a weakened cell wall was indicated by increased epidermal cell permeability, as suggested by strong uptake of the neutral red dye (Figure 6, NI1, NI2, top panel on the right side). In comparison, epidermal cell permeability did not increase in resistant Molly plants following Hessian fly attack (Figure 6, MI1, MI2), suggesting that cell wall and cuticle were strengthened in resistant plants after Hessian fly infestation and larvae were unable to increase cell wall permeability in these plants.

## Discussion

**Figure 7:**
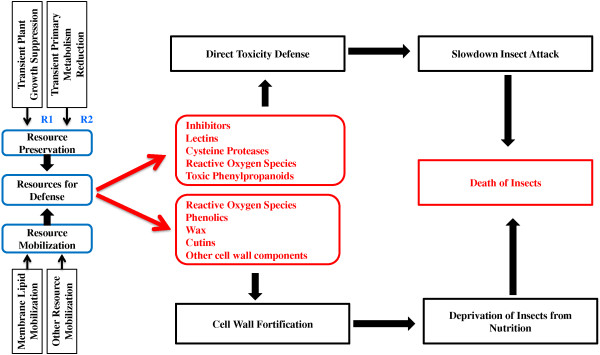
**A model for defense in resistant wheat plants against Hessian fly infestation.** Defense requires resources. Growth of resistant plants is transiently suppressed following Hessian fly infestation (R1: reference [29]); and primary metabolism in resistant wheat is also transiently suppressed after Hessian fly attack to preserve resources for defense (R2: reference [11]). Host resources including lipids, carbohydrates, and protein/amino acids are rapidly mobilized through catabolic processes in resistant plants in response to Hessian fly attack (Additional file 5: Table S4). The resources are used mainly for two types of defense, producing chemicals that are directly toxic to Hessian fly larvae, and substances that fortify cell walls. Toxic chemicals include various inhibitors of Hessian fly digestive enzymes, lectins, cysteine proteases, reactive oxygen species, and toxic secondary metabolites. The toxic chemicals may not be able to kill Hessian fly larvae alone, but can slow down insect attacking, which can allow plants more time to enhance other types of defense. Fortified cell walls prevent Hessian fly larvae from delivering effectors into host cells, and therefore, unable to induce the formation of nutritive cells. Failure of nutritive cell formation results in the death of Hessian fly larvae due to the lack of nutrition.

In this study, we analyzed changes in gene expression at multiple times following Hessian fly infestation in two nearly-isogenic wheat lines, susceptible Newton and its resistant backcross-offspring Molly [47]. A large number of transcripts exhibited significant changes in both resistant and susceptible wheat plants. In-depth analyses indicated that the gene sets in resistant plants affected by Hessian fly were quite different from those affected in susceptible plants, and early response genes were different from late response genes. Comparative analyses of the dynamic differences in gene expression in the resistant and susceptible wheat lines indicated that rapid mobilization and re-utilization of resources, enhanced direct toxic defense, and fortification of cell walls are coordinated defense processes that may be crucial for Hessian fly resistance in wheat (Figure 7).

### Preservation and rapid mobilization of resources for defense in infested resistant plants

In plants with an effective R gene, plant growth is transiently suppressed following Hessian fly larval attack [29]. The primary metabolic pathways were also transiently suppressed in resistant plants following Hessian fly infestation [11]. This transient suppression of plant growth and primary metabolism are likely an important step to preserve resources to launch plant defense (Figure 7). A great proportion of transcripts encoding enzymes in catabolic pathways, including lipases, lipid transfer proteins and a range of other transporters, sugar degradation enzymes, and amino acid degradation enzymes, were up-regulated with great magnitudes specifically in infested resistant plants (Additional file 6: Figure S2, Additional file 5: Table S4). The prompt up-regulation of catabolic enzymes indicates a prompt increase in degradation metabolism to release substances and energy needed for defense. The strong up-regulation of a large number of lipase and lipid transfer protein genes indicate that membrane remodeling and subsequent release of membrane lipids may be crucial to provide resources for defense. Mobilized lipids can be easily converted into defense molecules such as oxylipins, wax, and cutin components with small chemical modifications [35,36,48]. Degradation metabolism of lipids can also provide high energy for defense biosynthesis [11]. In consistent with the up-regulation of lipid mobilization genes, the abundance of various membrane lipids decreased, whereas a range of fatty acids and derivatives increased in resistant plants following Hessian fly attack (Figure 3 and Additional file 7: Table S5), indicating that the mobilized membrane lipids were indeed in the process to be converted into molecules for cell wall and cuticle strengthening and the launch of other types of defenses. In addition to mobilization of lipids, other substances including sugars, proteins, and amino acids are also likely mobilized (Additional file 5: Table S4). The mobilization of membrane lipids and other nutrients is consistent with a sharp decrease in nutrient metabolism in resistant plants following Hessian fly attack [11], a metabolic cost for plants to launch chemical defense.

### Direct toxic defense may slow down larval attack, but does not cause immediate lethality

A large number of transcripts encoding toxic proteins, including inhibitors of insect digestive enzymes, lectins, and cysteine proteases were up-regulated or exhibited higher levels of abundance in infested resistant plants (Figure 4, Additional file 3: Table S3), indicating that direct defense may play an important role in wheat defense against Hessian fly infestation. In addition to proteins that are directly toxic to Hessian fly, several types of transcripts encoding enzymes that can produce toxic chemicals were also up-regulated, including oxalate oxidases and class III peroxidases (Figure 4, Additional file 3: Table S3, Additional file 5: Table S4), both of which can produce reactive oxygen species [49-53]; and enzymes in the phenylpropanoid pathways, which can produce toxic secondary metabolites. The broad range of the up-regulated toxins indicates a possible strong effect of direct defense on Hessian fly larvae. However, the exact effect of toxic chemicals in plant resistance remains to be delineated. A rescue assay indicated that toxicity from defense chemicals in infested resistant plants did not translate into immediate lethality of Hessian fly larvae (Figure 4B). This observation is consistent with a recent report that virulent larvae can rescue avirulent larvae in otherwise resistant plants within three days after hatching [54]. Even though toxic chemicals alone could not kill Hessian fly larvae immediately, the chemicals may slow down larval attack [55], and therefore, allow more time for the plants to launch or enhance other types of defenses. Potential long term effect of toxins on Hessian fly development and reproduction remains to be determined.

### Rapid remodeling and fortification of cell walls may make fly larvae starve to death

Since toxicity alone did not kill Hessian fly larvae, additional defense mechanism(s) must be in play for the observed insect antibiosis in resistant wheat. Rapid remodeling and fortification of cell walls could play a key role by preventing Hessian fly larvae from accessing nutrients, causing the insects to die. Various types of transcripts encoding enzymes and proteins potentially involved in cell wall and cuticle strengthening are promptly and highly up-regulated specifically in resistant plants after larval attack. These proteins and enzymes, including expansins, xyloglucan transglycosylases, xyloglucan fucosyltransferases, glucanases, pectineste-rases, and Cer1 proteins, participate in various processes in cell wall loosening, remodeling, and fortification (Figure 6). In addition, increased production of various phenolics produced as a result of elevated gene expression in the phenylpropanoid pathway could have been deposited into the cell wall for strengthening (Figure 5) [56,57]. A large number of transcripts encoding class III perxoidases are quickly up-regulated in resistant wheat following Hessian fly attack (Additional file 9: Figure S3, Additional file 3: Table S3) [34]. Class III peroxidases can produce reactive oxygen species at extracellular space in response to parasite attack [50]. Elevated levels of reactive oxygen species can enhance cross-linking of deposited phenolics, resulting in fortified cell walls. Furthermore, portion of mobilized membrane lipids could have been converted into wax and cutins [35], providing additional cell impermeability in infested resistant plants. Consistent with above observations, our data with neutral red staining demonstrated that Hessian fly larvae were unable to increase cell wall permeability in infested resistant plants, suggesting that cell wall and cuticle were indeed strengthened in these plants. Fortified cell walls could prevent Hessian fly larvae from delivering effectors into wheat cells, preventing the insect from manipulating normal host tissue into nutritive tissues [30]. The lack of nutritive tissues prevents Hessian fly larvae from obtaining nutrition from host cells in resistant plants, resulting in their death.

## Conclusions

In summary, resistant wheat plants undergo rapid and coordinated responses to mobilize resources through remodeling cell membranes and likely other cellular structures to release substances and energy needed for plant defense in response to Hessian fly infestation (Figure 7). The mobilized resources are likely converted into toxic molecules, which may slow down insect attack and give plants more time to effectively launch or enhance other defense mechanisms. A swift remodeling and fortification of cell walls and cuticle may prevent Hessian fly larvae from delivering effectors into host cells, thus inhibiting nutritive tissue formation. The lack of nutritive tissue at the feeding site prohibits Hessian fly larvae from obtaining host nutrients, resulting in insect death due to malnutrition. Susceptible wheat is unable to quickly mobilize resources or convert them into defense molecules. As a result, plants are being manipulated by Hessian fly larvae, resulting in the formation of plant nutritive tissue and thriving of insects. Further research is needed to elucidate how a resistance protein detects Hessian fly larval attack, and promptly initiates signaling for the coordinated defense reactions as observed in this study.

## Methods

### Plant and insect materials

The wheat cultivars Newton and Molly were used in this study. Newton is a susceptible cultivar containing no Hessian fly resistance gene. Molly is a nearly-isogenic offspring line of Newton and contains the R gene *H13*. Molly was obtained via backcrossing for seven generations to the susceptible parent Newton [47]. A Hessian fly population from Scott County, Kansas, was used for this research. The fly population is virulent to Newton wheat, but avirulent to Molly, which contains the resistance gene *H13*[58].

### Experimental treatments and RNA extraction

Molly and Newton seedlings were grown and infested with biotype GP in a growth chamber set at 20 ± 1°C (daytime) and 18 ± 1°C (night) with a 14:10 h (L/D) photoperiod. Seedlings were infested at two-leaf stage with eggs from 20 mated females per 10 plants. Tissue samples from 5 plants in each replicate were collected and pooled at 6, 12, 24, and 72 h after larval hatching. Only the tissue at the feeding site (about 1.5 to 2 cm) of the second leaf sheath was collected for RNA extraction. Seedlings of each genotype under the same conditions but without infestation were used as uninfested control. A portion of the seedlings in each experiment was kept for later phenotypic confirmation. Total RNA was extracted from wheat tissues using TRI reagent™ according to the procedure provided by the manufacturer (Molecular Research Center, Inc., Cincinnati, OH, USA). Three biological replications were used for each treatment.

### Microarray hybridization and data analysis

GeneChip^®^ wheat genome arrays containing 61,127 probesets were purchased from Affymetrix (Santa Clara, CA, USA). Each data point contains three biological replicates. Labeling and hybridization on the wheat microarrays were performed according to the standard protocol provided by Affymetrix (http://www.affymetrix.com/support/technical/manual/expression_manual.affx). After hybridization and washing, the arrays were scanned with an Affymetrix GeneChip Scanner 3000. Gene annotation was accomplished using HarvEST: Wheat version 1.57 (http://www.harvest.ucr.edu/) and Affymetrix NetAffx. The functions of the genes were further manually updated by searching the GenBank with BLASTX. Microarray data were deposited to the data base of National Center for Biotechnology Information (NCBI) with accession number GSE34445 (http://www.ncbi.nlm.nih.gov/geo/query/acc.cgi?acc=GSE34445).

### Statistical analysis

The microarray probe-level intensity was read from cel files directly. The probe-level data from multiple chips were then summarized with GC Robust Multi-array Average (GCRMA) [59] to produce probeset expression values. GCRMA uses probe affinity data including perfect match, mismatch probes and sequence information to perform background adjustment, quantile normalization, and median-polish summarization.

Differentially expressed genes were identified by using LIMMA method. Specifically, the effects of interest were coded into contrast parameters and linear models were fitted to estimate the contrast parameters. The log-odds of differential expression versus no differential expression and the moderated t-statistics were calculated by empirical Bayes shrinkage of the standard errors towards a pooled standard deviation value. Differentially expressed genes were selected at 0.05 significant level with BH adjustment for multiple comparisons [60,61].

### Real-time PCR validation and correlation Analysis

Real-time PCR (qPCR) was carried with six representative genes for validation of microarray results. Actin was used for normalization. Total RNA was treated with TURBO™ DNase (Ambion, Austin, TX, USA) to remove any genomic DNA contamination. One microgram of total RNA was used for synthesis of first strand cDNA using SuperScript^®^ III First-Strand Synthesis System (Invitrogen, Carlsbad, CA, USA), and used as a template for real-time quantitative PCR (qPCR). Three biological replications, each with two technical replications were used for qPCR analysis. PCR primers were designed using Beacon Designer software (version 7) and listed in Additional file 10: Table S7. qPCR was performed using SYBR green kit (Bio-Rad) and Bio-Rad iCycler iQs real-time PCR detection system at the Kansas State University Gene Expression Facility. qPCR cycling parameters included 95°C for 5 min, 40 cycles each consisting of 95°C for 30 sec, 52°C for 15 sec, and 72°C for 45 sec. At the end of each PCR reaction, a melt curve was generated to confirm single peak and rule out the possibility of primer-dimer and non-specific product formation. Relative fold-changes for transcripts were calculated using the comparative 2^−ΔΔCT^ method [62]. Log2 ratios were used to perform the correlation analysis between qPCR and microarray data.

qPCR results are shown in Additional file 9: Figure S3A. The results were highly consistent with microarray results, with a correlation coefficient 0.95 (Additional file 9: Figure S3B).

### Hessian fly larvae rescue assay

Initial infestation was carried out with Molly plants as described above. Larval hatching and migration were monitored hourly starting three days after infestation to accurately determine the time when larvae reached the feeding site [36]. After larvae lived in resistant Molly plants for one or two days, seedlings were dissected, larvae in the plants were washed off into water with 0.1% Tween20. The washed off larvae were individually replaced back onto the first leaf-blade of a fresh seedling of the susceptible Newton. Survival rate of the rescued larvae was determined two weeks later when larvae became third instar. For control, larvae lived in susceptible Newton seedlings for one or two days were collected and transferred onto new Newton seedlings in exactly the same way as in Molly. Five replicates were carried out and each replicate consisted of 10 plants with average rescued larvae 20–30 per replicate. Data were analyzed using a standard ANOVA analysis.

### Metabolite profiling

Metabolite profiling was conducted by a commercial service with Metabolon (Durham, NC, http://www.metabolon.com/). The analysis was conducted as described previously [63]. Briefly, wheat tissues were processed using the automated MicroLab STAR^®^ system from Hamilton Company (Reno, NV, USA). Recovery standards were added prior to the first step in the extraction process for quality control purposes. Sample preparation was conducted using a proprietary series of organic and aqueous extractions to remove the protein fraction while allowing maximum recovery of small molecules. The resulting extract was divided into two fractions; one for analysis by liquid chromatography (LC) and the other for analysis by gas chromatography (GC). Samples were placed briefly on a TurboVap^®^ (Zymark) to remove the organic solvent. Each sample was then frozen and vacuum dried. Samples were then subjected to mass spectrometry (MS), either LC/MS or GC/MS.

LC/MS analysis was conducted on a platform based on a Waters ACQUITY UPLC and a Thermo-Finnigan LTQ mass spectrometer, which consisted of an electrospray ionization (ESI) source and a linear ion-trap (LIT) mass analyzer. Plant extract was split into two aliquots, dried, then reconstituted in acidic or basic LC-compatible solvents, each of which contained 11 or more injection standards at fixed concentrations. One aliquot was analyzed using acidic positive ion optimized conditions and the other using basic negative ion optimized conditions in two independent injections using separate dedicated columns. Extracts reconstituted in acidic conditions were gradient eluted using water and methanol both containing 0.1% Formic acid, while the basic extracts, which also used water/methanol, contained 6.5 mM ammonium bicarbonate. The MS analysis alternated between MS and data-dependent MS^2^ scans using dynamic exclusion.

The samples destined for GC/MS analysis were re-dried under vacuum desiccation for a minimum of 24 hours prior to being derivatized under dried nitrogen using bistrimethyl-silyl-triflouroacetamide (BSTFA). The GC column was 5% phenyl and the temperature ramp is from 40° to 300°C in a 16 minute period. Samples were analyzed on a Thermo-Finnigan Trace DSQ (Fisher scientific, Pittsburgh, PA) fast-scanning single-quadrupole mass spectrometer using electron impact ionization.

Data were collected and analyzed using an informatics system. Compounds were identified by comparison to library entries of purified standards or recurrent unknown entities. Identification of known chemical entities was based on comparison to metabolomic library entries of purified standards, which consisted of over 1000 commercially available purified standard compounds. The combination of chromatographic properties and mass spectra gave an indication of a match to the specific compound or an isobaric entity. A variety of curation procedures were carried out to ensure that a high quality data set was made available for statistical analysis and data interpretation.

*t*-tests were conducted to determine whether the unknown means for two populations are different or not. “Mean Decrease Accuracy” (MDA) is used to determine which variables (biochemicals) make the largest contribution to a classification. MDA is determined by randomly permuting a variable, running the observed values through the trees, and then reassessing the prediction accuracy. Thus, the random forest analysis provides an “importance” rank ordering of biochemicals; we typically output the top 30 biochemicals in the list as potentially worthy of further investigation.

### Epidermal cell permeability assay

Neutral red stain (Sigma-Aldrich, St. Louis, MO, USA) was used to determine epidermal permeability of cells as described previously [35,36]. Briefly, plants were dissected 2 days after the initial HF larval attack. After peeling off the first leaf-sheath, the HF larval feeding site of the second leaf-sheath was stained with 0.1% neutral red stain for 10 min, followed by five times washing with water. Uninfested plants were dissected and stained as negative controls in the same way. After staining, plant tissues were examined under a fluorescent microscope (Zeiss Axioplan-2) and photographed with a Nikon Coolpix 4500 Digital camera.

## Competing interests

The authors declare that they have no competing interests.

## Authors’ contributions

CK, XL and SW performed experiments. CK, HW, and MSC analyzed data, JCR and RJW contributed reagents and edited the manuscript, MEB analyzed data and edited the manuscript, MSC and CK designed the experiment and wrote the paper. All authors read and approved the final manuscript.

## Supplementary Material

Additional file 1: Table S1 Probe sets that detected significant changes in transcript abundance at *P* ≤ 0.01. Click here for file

Additional file 2: Table S2 Venn diagrams showing the numbers of common probe sets that detected changes in different combinations of treatments or time points. Click here for file

Additional file 3: Table S3 Fold change and P-values of the 10 categories of transcripts. Molly is a Hessian fly resistant wheat cultivar and Newton is a susceptible cultivar. Click here for file

Additional file 4: Figure S1 Defense genes are generally expressed at high levels in resistant plants than in susceptible plants following Hessian fly attack. Hessian fly-affected genes with known functions were classified into 11 categories based on gene annotation, including genes involved in direct toxic defense (direct defense), genes involved in lipid metabolism (lipid metabolism), genes involved in phenylpropanoid metabolism (phenylpropanoid), genes involved in cell wall metabolism (cell wall), genes involved in oxidation (redox), genes encoding proteases (proteases), genes involved in regulation (regulation), genes encoding structural proteins (structure), genes involved in nutrient metabolism (nutrition), and genes involved in stress response (stress response). Incompatible/compatible ratio (I/C ratio) were defined as the ratio of fold changes in resistant plants during incompatible interactions against fold changes in susceptible plants during compatible interactions after Hessian fly infestation. Red arrows on the left side of each graph indicates the crossing line, above which means a higher transcript level in infested resistant plants, whereas below which means a higher transcript level in infested susceptible plants. Transcripts with I/C ratio more than 20 were represented by 20 to reduce the complexity of the graphs. Click here for file

Additional file 5: Table S4 Genes potentially involved in resources mobilization. Transcripts that may have similar functions are highlighted in the same color. Click here for file

Additional file 6: Figure S2 Resource mobilization genes are up-regulated rapidly in infested resistant plants. **A**: Genes involved in lipid catabolism (Additional file 5: Table S4). (**a**) Percentages of lipid-related genes that were up- (darker bar) or down-regulated (lighter bar) in plants during incompatible (I) and compatible (C) interactions. (**b**) Average fold changes of lipid-related genes at different time points after Hessian fly infestation. IU, ID, CU, and CD represent fold changes of up-regulated transcripts during incompatible interaction, of down-regulated transcripts during incompatible interactions, of up-regulated transcripts during compatible interactions, and of down-regulated transcripts during compatible interactions. **B**: Genes encoding other types of transporters. **C**: Genes encoding carbohydrate and protein/amino acid catabolic enzymes. **D**: Genes encoding various anabolic enzymes. Click here for file

Additional file 7: Table S5 Evidence of lipid mobilization and membrane remodeling from changes of metabolites. Click here for file

Additional file 8: Table S6 Changes in abundance of transcripts encoding proteins that are toxic to insects or enzymes that can produce toxins. Click here for file

Additional file 9: Figure S3 Validation of microarray data through qPCR. A: qPCR results of six representative genes with GenBank accession numbers CK213159, CN009367, CD869243, BQ295073, CD875175, and BQ838257. B: Correlation analysis of microarray and Real-time PCR data sets. Click here for file

Additional file 10: Table S7 Target genes and primer pairs used for real-time PCR (qPCR). Click here for file
