# Supplementary material for: Mobilization of lipids and fortification of cell wall and cuticle are important in host defense against Hessian fly
Source: BMC Genomics. 2013 Jun 26;14:423. doi: 10.1186/1471-2164-14-423 (PMC3701548; doi:10.1186/1471-2164-14-423)
Supplement: Additional file 4: Figure S1 — Defense genes are generally expressed at high levels in resistant plants than in susceptible plants following Hessian fly attack. Hessian fly-affected genes with known functions were classified into 11 categories based on gene annotation, including genes involved in direct toxic defense (direct defense), genes involved in lipid metabolism (lipid metabolism), genes involved in phenylpropanoid metabolism (phenylpropanoid), genes involved in cell wall metabolism (cell wall), genes involved in oxidation (redox), genes encoding proteases (proteases), genes involved in regulation (regulation), genes encoding structural proteins (structure), genes involved in nutrient metabolism (nutrition), and genes involved in stress response (stress response). Incompatible/compatible ratio (I/C ratio) were defined as the ratio of fold changes in resistant plants during incompatible interactions against fold changes in susceptible plants during compatible interactions after Hessian fly infestation. Red arrows on the left side of each graph indicates the crossing line, above which means a higher transcript level in infested resistant plants, whereas below which means a higher transcript level in infested susceptible plants. Transcripts with I/C ratio more than 20 were represented by 20 to reduce the complexity of the graphs. [file 1471-2164-14-423-S4.pptx]

## Slide 1
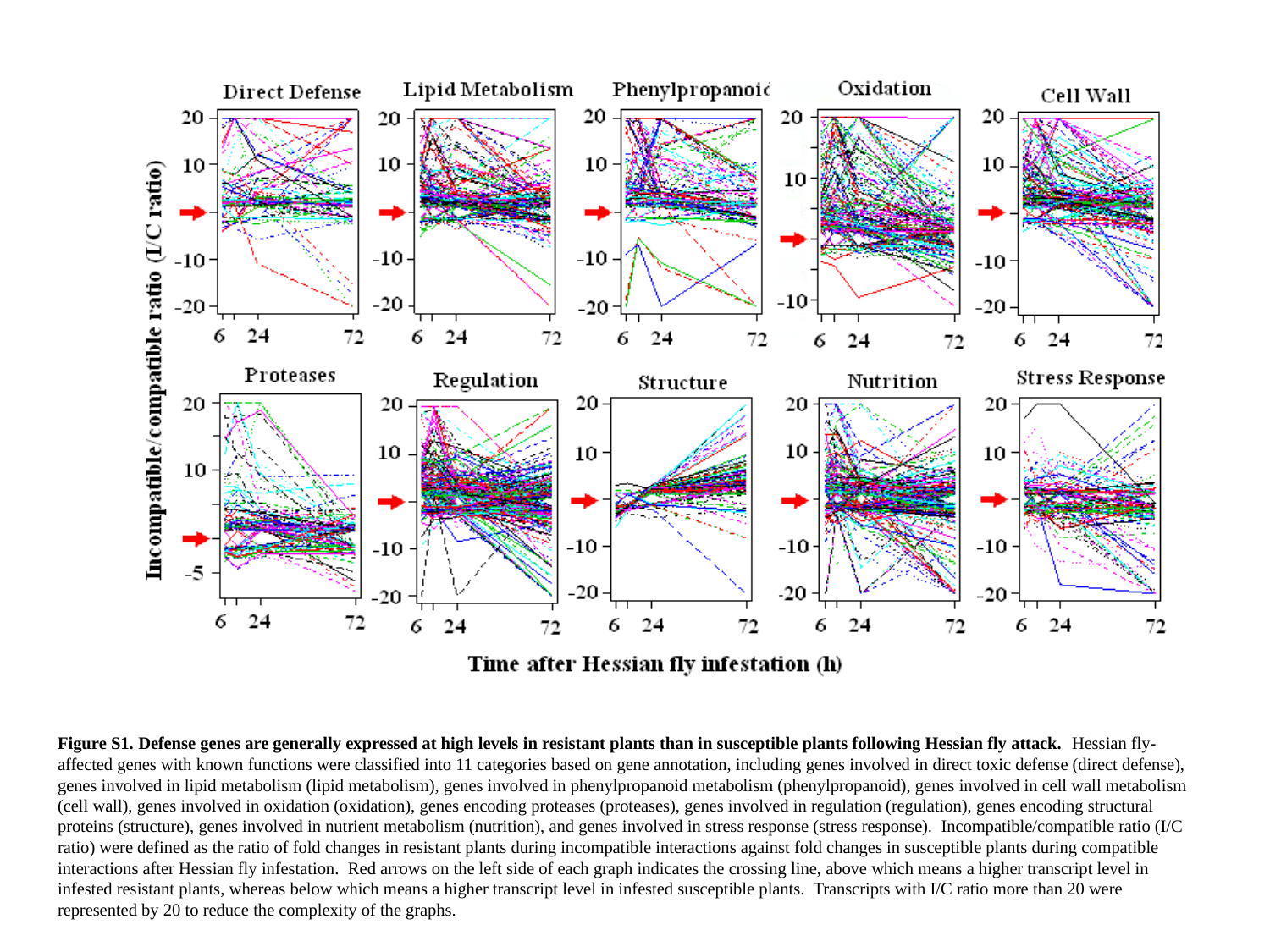

Figure S1. Defense genes are generally expressed at high levels in resistant plants than in susceptible plants following Hessian fly attack. Hessian fly-affected genes with known functions were classified into 11 categories based on gene annotation, including genes involved in direct toxic defense (direct defense), genes involved in lipid metabolism (lipid metabolism), genes involved in phenylpropanoid metabolism (phenylpropanoid), genes involved in cell wall metabolism (cell wall), genes involved in oxidation (oxidation), genes encoding proteases (proteases), genes involved in regulation (regulation), genes encoding structural proteins (structure), genes involved in nutrient metabolism (nutrition), and genes involved in stress response (stress response). Incompatible/compatible ratio (I/C ratio) were defined as the ratio of fold changes in resistant plants during incompatible interactions against fold changes in susceptible plants during compatible interactions after Hessian fly infestation. Red arrows on the left side of each graph indicates the crossing line, above which means a higher transcript level in infested resistant plants, whereas below which means a higher transcript level in infested susceptible plants. Transcripts with I/C ratio more than 20 were represented by 20 to reduce the complexity of the graphs.
