# Supplementary material for: Mobilization of lipids and fortification of cell wall and cuticle are important in host defense against Hessian fly
Source: BMC Genomics. 2013 Jun 26;14:423. doi: 10.1186/1471-2164-14-423 (PMC3701548; doi:10.1186/1471-2164-14-423)
Supplement: Additional file 6: Figure S2 — Resource mobilization genes are up-regulated rapidly in infested resistant plants. A: Genes involved in lipid catabolism (Additional file 5: Table S4). (a) Percentages of lipid-related genes that were up- (darker bar) or down-regulated (lighter bar) in plants during incompatible (I) and compatible (C) interactions. (b) Average fold changes of lipid-related genes at different time points after Hessian fly infestation. IU, ID, CU, and CD represent fold changes of up-regulated transcripts during incompatible interaction, of down-regulated transcripts during incompatible interactions, of up-regulated transcripts during compatible interactions, and of down-regulated transcripts during compatible interactions. B: Genes encoding other types of transporters. C: Genes encoding carbohydrate and protein/amino acid catabolic enzymes. D: Genes encoding various anabolic enzymes. [file 1471-2164-14-423-S6.pptx]

## Slide 1
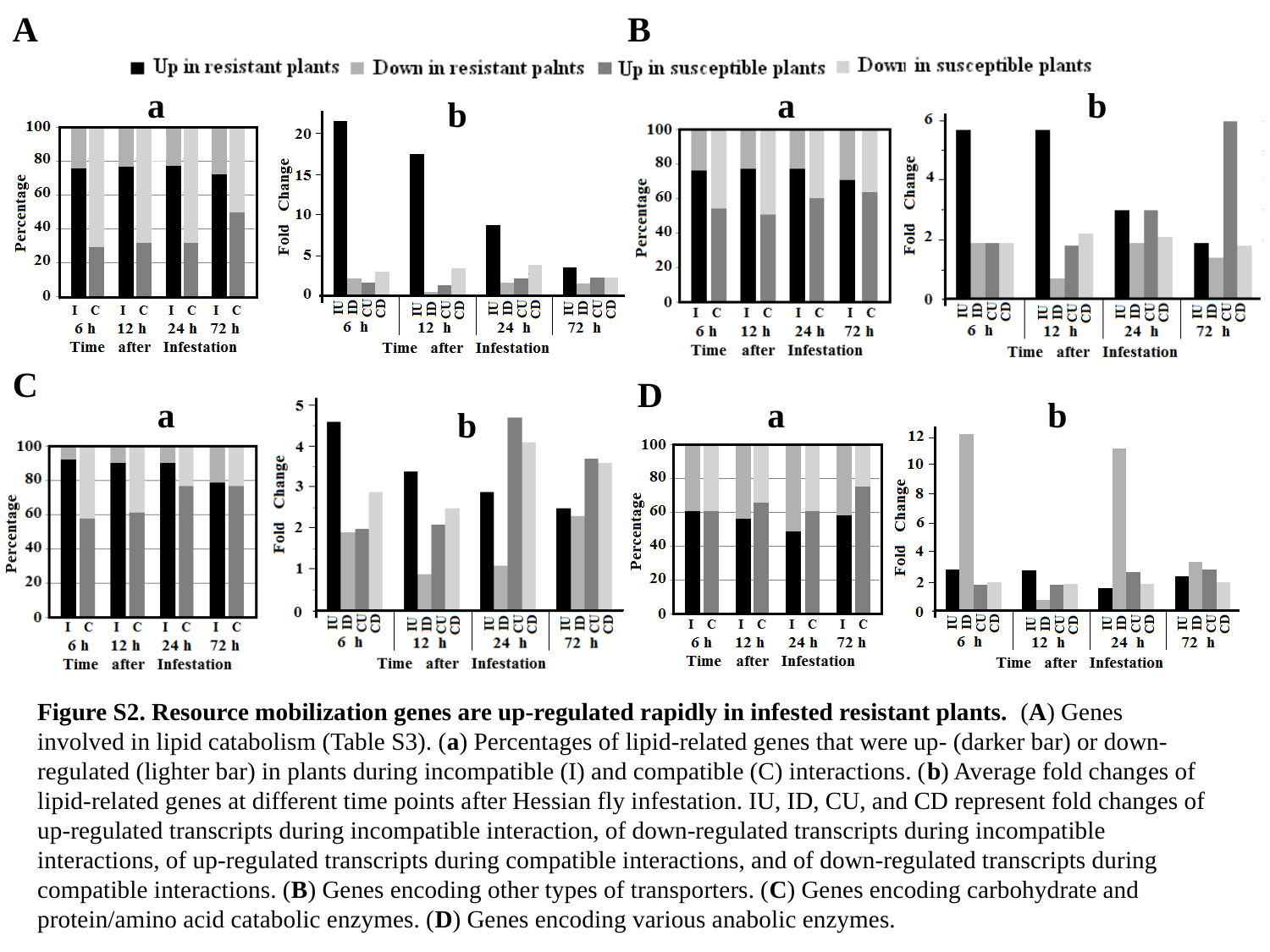

A
B
a
a
b
b
C
D
a
a
b
b
Figure S2. Resource mobilization genes are up-regulated rapidly in infested resistant plants. (A) Genes involved in lipid catabolism (Table S3). (a) Percentages of lipid-related genes that were up- (darker bar) or down-regulated (lighter bar) in plants during incompatible (I) and compatible (C) interactions. (b) Average fold changes of lipid-related genes at different time points after Hessian fly infestation. IU, ID, CU, and CD represent fold changes of up-regulated transcripts during incompatible interaction, of down-regulated transcripts during incompatible interactions, of up-regulated transcripts during compatible interactions, and of down-regulated transcripts during compatible interactions. (B) Genes encoding other types of transporters. (C) Genes encoding carbohydrate and protein/amino acid catabolic enzymes. (D) Genes encoding various anabolic enzymes.
