# Supplementary material for: Mobilization of lipids and fortification of cell wall and cuticle are important in host defense against Hessian fly
Source: BMC Genomics. 2013 Jun 26;14:423. doi: 10.1186/1471-2164-14-423 (PMC3701548; doi:10.1186/1471-2164-14-423)
Supplement: Additional file 9: Figure S3 — Validation of microarray data through qPCR. A: qPCR results of six representative genes with GenBank accession numbers CK213159, CN009367, CD869243, BQ295073, CD875175, and BQ838257. B: Correlation analysis of microarray and Real-time PCR data sets. [file 1471-2164-14-423-S9.pptx]

## Slide 1
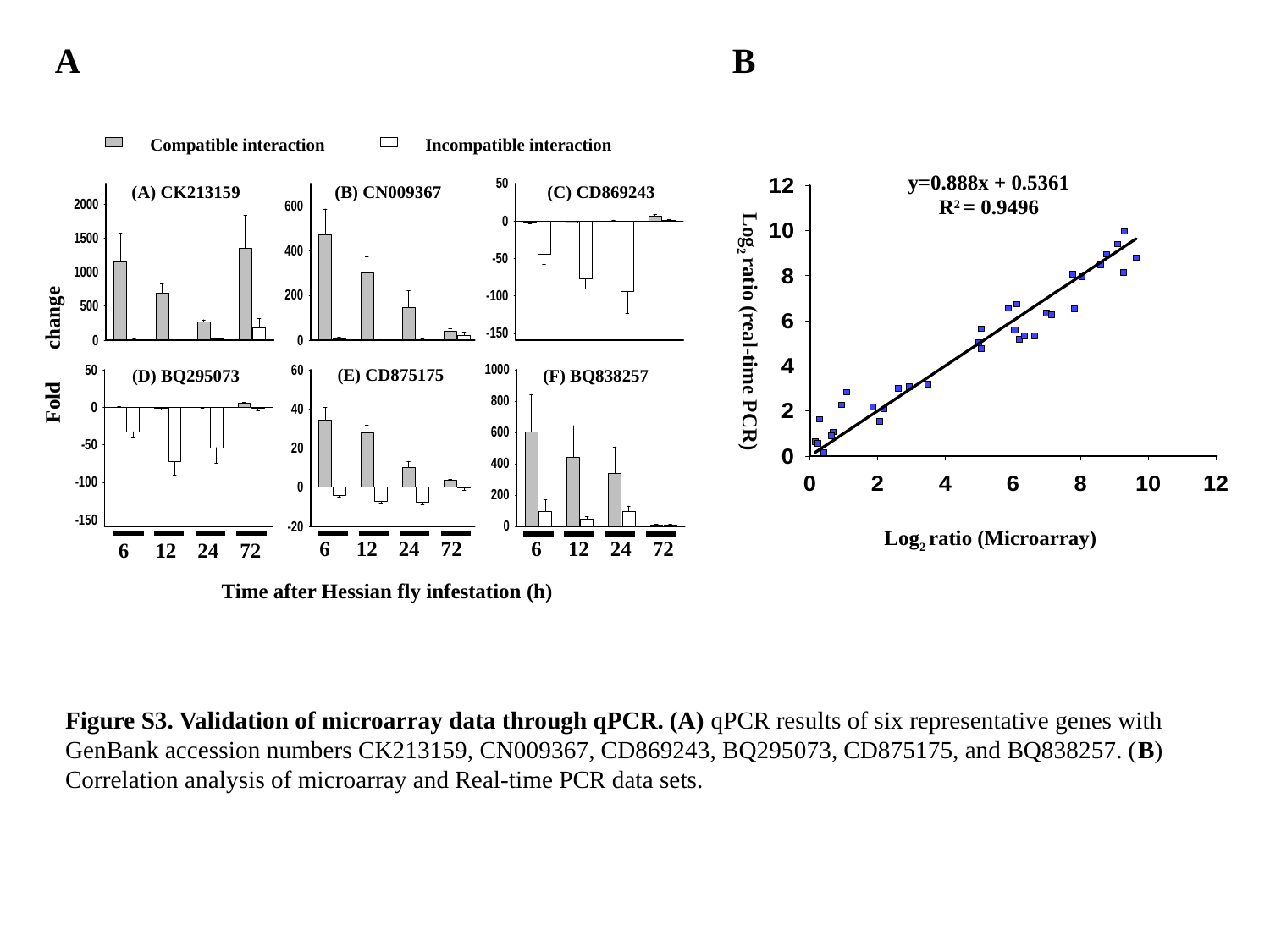

A
B
Compatible interaction
Incompatible interaction
(A) CK213159
(B) CN009367
(C) CD869243
Fold change
(E) CD875175
(D) BQ295073
(F) BQ838257
 6 12 24 72
y=0.888x + 0.5361
R2 = 0.9496
Log2 ratio (real-time PCR)
Log2 ratio (Microarray)
 6 12 24 72
 6 12 24 72
Time after Hessian fly infestation (h)
Figure S3. Validation of microarray data through qPCR. (A) qPCR results of six representative genes with GenBank accession numbers CK213159, CN009367, CD869243, BQ295073, CD875175, and BQ838257. (B) Correlation analysis of microarray and Real-time PCR data sets.
