# Supplementary material for: Mobilization of lipids and fortification of cell wall and cuticle are important in host defense against Hessian fly
Source: BMC Genomics. 2013 Jun 26;14:423. doi: 10.1186/1471-2164-14-423 (PMC3701548; doi:10.1186/1471-2164-14-423)
Supplement: Additional file 10: Table S7 — Target genes and primer pairs used for real-time PCR (qPCR). [file 1471-2164-14-423-S10.doc]

**Table S7. Target genes and primer pairs used for real-time PCR (qPCR).**

| Sequence name | Gene ID | PL* (bp) | Primer Sequence |
| --- | --- | --- | --- |
| O-methyltransferase | CD875175 | 93 | F: ATT AAC AAT ACA GAC AGT TCA TG |
|  |  |  | R: TAC TAA TGG ACA CCC TAC C |
| Peroxidase 53 | BQ295073 | 79 | F: AGA CAA CGT GTG GGT ATA |
|  |  |  | R: TTA CAC CAG AGA CGA CAA |
| Germin-like protein | CD869243 | 151 | F: CAC CAG CAC ATT ATG ACA T |
|  |  |  | R: TGG GCA TAT GGG AAG TTA |
| WRKY45 transcription factor | BQ838257 | 75 | F: GTA GAC GGT AGT GCT GTG |
|  |  |  | R: ATG GAC ATC TCG TGA ACT |
| 12-oxo-phytodienoic acid reductase | CN009367 | 76 | F: CAG ACT CAA ACC CAG AAG |
|  |  |  | R: ATG TGG CAG TAG AGG ATT |
| Lipoxygenase | CK213159 | 135 | F: CAG CTT CAT CAA CAG GAA |
|  |  |  | R: TGT TAT GGC GTT ATG TTA TTG |
| Actin | AF326781 | 127 | F: AAA TCT GGC ATC ACA CTT TCT AC |
|  |  |  | R: GTC TCA AAC ATA ATC TGG GTC ATC |

*Product length; F: Forward primer; R: Reverse primer
